# Methods Used to Support Dietary Assessment Accuracy in Young Children: A Scoping Review

**DOI:** 10.3390/nu18213631

**Published:** 2026-10-31

**Authors:** Emilie Holloway Pratt, Elizabeth Anderson Steeves, Katie Kavanagh, Angela Fidler Pfammatter, Niki Cobb, Hollie A. Raynor

**Affiliations:** 1 Department of Nutrition and Public Health Sciences, The University of Tennessee-Knoxville, Knoxville, TN 37996, USA; kkavanag@utk.edu (K.K.); angela@utk.edu (A.F.P.); hraynor@utk.edu (H.A.R.); 2 Center for Nutrition and Health Impact, Omaha, NE 68022, USA; easteeves@centerfornutrition.org; 3 College of Education, Health, and Human Sciences, The University of Tennessee-Knoxville, Knoxville, TN 37996, USA; 4 University Libraries, The University of Tennessee-Knoxville, Knoxville, TN 37996, USA; niki@utk.edu

**Keywords:** children, dietary assessment, energy intake, methodology

## Abstract

Background/Objectives: Among young children, self-reported dietary assessment has unique challenges, limiting ability to accurately assess intake. This review describes methods used to support accuracy of self-reported dietary intake, including support tools, proxy reporting, and methods to determine diet misreporting and its prevalence, for assessing energy intake (EI) among young children (2–12 years). Methods: Following PRISMA-ScR guidelines, relevant databases were searched in October 2024 and November 2025. Studies published since 2000, using a self-reported dietary assessment methodology and reporting on total EI among children between the ages of 2 and 12 years, were included. Two reviewers completed title and abstract screening. After achieving a 94% inter-rater agreement on full-text review of a random sample of 20% of texts, the remaining full texts were reviewed by a single reviewer. Results: The 52 included articles reported 24-h dietary recalls (*n* = 23) and food records (*n* = 14) as the most common individual assessment methods. The use of at least one support tool (i.e., visual aids, household utensils) was commonly reported (*n* = 36); however, using multiple support tools (*n* = 13) or technology-based supports (*n* = 1) was less common. Proxy reporting was frequently reported (*n* = 31) and occurred in all studies in which children were ≤ 5 years; however, few studies used multiple-proxy reporters (*n* = 6). A limited number of studies (*n* = 9; 17%) reported that caregivers and children were trained for the dietary assessment, but none actually described in detail what the training entailed for using support tools or proxy reporting. Only 40% of studies reported procedures for identifying dietary misreporting; among those that did, methodologies varied, yet all identified evidence of dietary misreporting. Conclusions: Self-reported dietary assessment used varying strategies to support assessment accuracy. Evaluating which support tool, combination of support tools, or the use of proxy or multiple-proxy reporters, relative to the burden that may be associated with their use, on increasing accuracy of self-reported dietary intake is needed. Additionally, details on implemented methodologies need to be consistently reported to support replication.

## 1. Introduction

Dietary assessment is essential for understanding the relationships between diet and health outcomes and helps inform the development of nutrition policies and population-level dietary guidance. Before selecting an assessment methodology, the overall research questions, objectives, and available resources should be considered, as these factors will guide the selection of an appropriate method. Methodologies commonly used in research- and practice-based settings to estimate total energy intake (EI) include 24 h dietary recalls (24HR), food records (FRs), and food frequency questionnaires (FFQs) [1,2,3,4]. Across the lifespan, these methodologies are often criticized for their reliance on self-report and/or memory-based recall [2,5].

Dietary misreporting includes both over- and underestimation and may occur through intrusions, omissions, or inaccurately reported food descriptions or portion sizes. A literature review of EI misreporting among children and adolescents (<18 years) by Forrestal et al. [6] found variable prevalences of dietary misreporting, ranging from 2 to 85% for under-reporters and 3 to 46% for over-reporters. Among young children, one factor that may contribute to misreporting dietary intake is age-appropriate level of cognitive development, which can limit a child’s ability to engage in the dietary assessment process [7,8]. Younger ages may have difficulty recalling foods they have consumed due to limitations in memory processing, communication skills, literacy level, numeracy skills, and limited knowledge of food preparation [7,8]. In fact, research shows that children are unable to accurately or reliably estimate intake until around the age of eight [9]. However, for children over eight, accuracy of self-reported dietary intake has also been criticized, as while cognitive development (e.g., memory and communication) has improved, there may still be limited knowledge of food preparation, food descriptions, and portion quantification. These age-appropriate limitations in young children may contribute to inaccuracies in self-reported dietary assessment, leading to dietary misreporting.

The known prevalence of dietary misreporting and the challenges that lead to inaccuracies highlight the need to implement methods that support accurate assessment of dietary intake in children. For children under the age of eight, proxy reporting, often by a parent or caregiver, is one method frequently utilized [10]. However, with more than half of children in the United States (US) attending regular childcare away from their primary caregiver [11], single-proxy reporters may not be present to capture all foods their child consumes across the day, which may lead to incomplete or inaccurate reporting on consumption [10]. While less is known about the prevalence of dietary misreporting by proxies, a single study by Börnhorst et al. [12] used proxy reporting in a sample of 2- to 9-year-old children and found prevalences of 8% and 3.4% for over- and under-reporting, respectively. Other methods that may support accuracy of portion size estimation have been used in dietary assessment, and include 2-dimensional food reference booklets, food models, and household utensils (e.g., measuring cups and spoons, standardized bowls and plates), which may be used both by proxies and children. More recently, in samples of older children and adults, technological advancements such as mobile devices, wearable sensors, and food photography have been used to increase accuracy of dietary assessment [13,14,15,16].

To better understand existing methods used to support accurate assessment of self-reported dietary intake in young children, this scoping review describes current methodologies utilized in self-reported dietary assessment that captures total EI among young children (aged 2–12 years) in studies published since 2000. Unlike a systematic review, which seeks to answer focused research questions through evidence synthesis and critical appraisal of the existing literature, a scoping review intends to map the existing body of literature by describing characteristics of the available evidence, which can help identify gaps for future research [17]. To our knowledge, no scoping review has comprehensively described the methods that have been used to support accurate assessment of dietary intake among young children. The review identifies factors including dietary assessment characteristics (i.e., types, settings, modalities), the use of support tools (i.e., visual aids, household utensils, etc.), proxy reporting, and methods used to detect dietary misreporting and its prevalence. Understanding gaps in current methods has the potential to inform future development of approaches that support accurate EI estimation when self-reported dietary assessment is used among young children.

## 2. Methods

### 2.1. Protocol and Registration

The protocol was drafted using the Preferred Reporting Items for Systematic Review and Meta-Analyses extension for Scoping Review (PRISMA-ScR) guidelines [18]. The final protocol was retrospectively registered on the Open Science Framework (https://doi.org/10.17605/OSF.IO/HQ2AY Accessed 5 August 2025), as at a later date it was realized that the registration process had not been fully completed. The registration was completed at that time.

### 2.2. Eligibility Criteria

#### 2.2.1. Participants

This scoping review describes approaches used to support accuracy of self-reported dietary assessment methods capturing total EI among children between the ages of 2 and 12 years at study initiation. Studies including a wider range of children (i.e., 6–18 years) were included as long as the mean age of the study sample fell within the specified range (2–12 years). When a mean age was not provided or insufficient detail was given, studies were included if the specified participant range included children within the 2–12 year range. Studies including children and other populations, including adults, were excluded if the primary objective of the study was not child specific. Studies that assessed EI only among infants (0–1 years), older children and adolescents (>12 years), or adults (>18 years) were excluded.

#### 2.2.2. Concept

All texts from academic authors mentioning self-reported dietary assessment methodologies capable of capturing total EI were included. Studies were excluded if they utilized an assessment methodology that could not capture or report on total EI, including but not limited to screeners and FFQs assessing individual food groups or nutrients. Additionally, non-dietary nutrition assessment methodologies were excluded, including eating behavior, malnutrition, and nutrition knowledge assessments.

#### 2.2.3. Context

This review includes texts from academic authors published since 2000 that were available in English. No other context limitations were enforced.

### 2.3. Information Sources and Search Strategy

To identify potentially relevant sources, the following databases were searched on 13 October 2024: PubMed (MEDLINE), Web of Science (WoS), SCOPUS, Embase, CINAHL (EBSCO), CABI (WoS), Cochrane Library, Education Source (EBSCO), and ERIC (EBSCO). Pilot searches within each database were conducted in early October 2024 to ensure the search terms were appropriate for the review questions. A second search was conducted on 18 November 2025, to identify articles published between October 2024 and November 2025.

Review team members (E.H.P. and H.R.) drafted search strategies with assistance from an experienced librarian (N.C.). Table 1 shows the final search strategy for each database; the same search strategy was used in October 2024 and November 2025. Final search results were imported to Covidence. In Covidence, duplicates were first removed and then all articles underwent screening and extraction with assistance from N.C.

### 2.4. Selection of Sources of Evidence and Data Charting

For both search periods (October 2024 and November 2025), two independent reviewers (E.H.P., R.H., and R.M.) screened titles and abstracts of all articles. To increase consistency, reviewers attended a training session where 30 titles and abstracts were randomly selected for review. Reviewers compared abstracts to a predefined list of inclusion and exclusion criteria, discussed selections and discrepancies, and modified the screening criteria before beginning independent reviews. When discrepancies arose, a third independent reviewer resolved conflicts, with discussion or feedback from other reviewers as needed.

For the first search period (October 2024) twenty percent of full texts (*n* = 30) were randomly selected for review by two independent reviewers (E.H.P., R.H., and R.M.). Then, two reviewers met to discuss inclusion and exclusion criteria and made modifications before beginning the review. During the first search period, an agreement of 94% was reached among the random sub-sample of articles. A priori, it had been decided that if there was at least 80% agreement between the two reviewers, the remaining full texts were reviewed by a single reviewer (E.H.P.) to determine inclusion. For the second search period, a single reviewer (E.H.P.) reviewed all articles for inclusion. Additional meetings with two reviewers (E.H.P. and H.A.R.) were held to confirm the articles selected for inclusion in the review.

### 2.5. Data Extraction Process and Items

A data extraction template was developed a priori. The extraction template included relevant information on participant characteristics (child and adult caregivers) and dietary assessment characteristics (i.e., type of assessment, settings, modality, use of support tools, proxy reporting, and dietary misreporting). The template was developed after pilot searches conducted before the start of the screening process. Modifications were made following full-text review of included studies based on pertinent information gathered during the screening process. The full extraction template is provided in Supplementary Materials.

A single reviewer (E.H.P.) extracted data in October 2024 and November 2025 using Covidence. To ensure accuracy of the extracted data, the reviewer re-reviewed all articles in August 2026. Two adjustments were made in the second review, one adjustment related to proxy reporting and one to dietary misreporting; however, these adjustments did not impact interpretation of the findings. After extraction, variables were transferred to an Excel table, where findings were summarized through descriptive and narrative synthesis. When a broad age range of children was included in the study, and the number of children within differing age subgroups was reported, these details were included in the table to support interpretation. Descriptive statistics (mean, median) of child ages are summarized for the overall sample when reported, or by subgroup when provided. For comparisons of the use of support tools (i.e., visual aids, household utensils, etc.), proxy-reporting, and the methods used for detecting dietary misreporting and its prevalence, data are summarized by life stage: only young children (≤5 years), only school-aged children (6–12 years), children aged ≥6 years, and mixed-age samples which included both young children (≤5 years), and school-aged children and above (≥6 years).

## 3. Results

### 3.1. Article Screening

A total of 6097 articles were identified through the search strategy (*n* = 5840 during the first search completed in October 2024, and *n* = 257 articles during the second search completed in November 2025). Table 2 summarizes the databases searched and the counts from each database. Following removal of duplicate sources (*n* = 2543), 3554 articles underwent title and abstract screening, with 145 moving to full-text screening. Figure 1 includes the PRISMA-ScR diagrams depicting the flow from article identification to selection. Following full-text review, 52 articles met eligibility criteria and are described in this review. A description of study, participant, and dietary assessment characteristics for all included studies can be found in Table 3.

### 3.2. Study Design, Settings, and Populations

The majority of the 52 included studies were published since 2012 (*n* = 33) [12,19,20,21,22,23,24,25,26,27,28,29,30,31,32,33,34,35,36,37,38,39,40,41,42,43,44,45,46,47,48,49,50]. The most commonly used study designs for assessing EI among children included cross-sectional (*n* = 30) [12,19,21,23,24,25,27,29,30,31,35,37,40,41,43,46,47,48,49,50,51,52,53,54,55,56,57,58,59,60], longitudinal or cohort designs (*n* = 7) [26,33,45,61,62,63,64], and randomized controlled trials (*n* = 6) [20,22,32,39,44,65]. Most studies occurred in North American (*n* = 22) [21,22,26,27,28,29,30,32,33,35,36,38,39,40,42,43,45,46,61,63,66,67] or European (*n* = 14) [12,20,23,31,37,44,50,53,56,57,58,60,62,64] countries. Regarding study settings, 45 studies conducted dietary assessments solely in free-living settings [19,21,22,23,24,25,26,27,28,29,30,32,33,34,35,37,39,40,41,42,43,44,45,46,47,48,50,51,52,53,54,55,56,58,59,60,61,62,63,64,65,66,67,68,69]. Several used a combination of settings, including free-living and lab-based (*n* = 2) [36,38] or free-living and school-based (*n* = 4) [12,20,31,57]. One study completed assessments among children with Type 1 Diabetes who were in acute care at the time of data collection [49].

The total number of participants ranged from 13 [66,67] to 9560 [12]. Broken down by life stage, seven studies assessed intake only among young children ≤ 5 years [21,22,23,26,29,33,68]. Thirty-eight percent of studies (*n* = 20) assessed intake among children only between the ages of 6 and 12 years [19,20,25,27,30,31,32,36,37,40,42,43,45,46,48,53,57,61,66,67]. The remaining studies were conducted among children aged 6 years and older (*n* = 10) [34,38,39,41,49,54,58,62,63,64] or among mixed-age samples (including children between the ages of 0 and 18 years (*n* = 15) [12,24,28,35,44,47,50,51,52,55,56,59,60,65,69].

### 3.3. Types and Modality of Assessment

Most studies used one method for assessing EI. The most common method was the 24HR (*n* = 23) [12,21,22,24,25,26,27,29,32,33,35,37,40,41,42,45,46,54,58,59,65,66,67] followed by FR (*n* = 14) [19,20,23,31,36,38,39,44,50,52,56,61,62,64] and FFQ (*n* = 7) [30,34,43,49,51,63,69]. Several studies used a combination of assessment methodologies, with the most frequent combination being 24HR paired with a FFQ (*n* = 6) [28,47,48,53,55,60]. One study by Ruel et al. [68] used a combination of methods including direct weighing of foods while children were in childcare, a recall approach for foods consumed outside of the direct weighing period, and a 48 h recall approach to capture weekend eating occasions.

**Table 3:** Summary characteristics of studies (*n* = 52) that assessed total energy intake using self-reported dietary assessment methodologies among children aged 2–12 Years.

Study Author, Year of Publication	Country	Study Design	Study Setting	Sample Size	Age, yrs M ± SD (Range)	Sample Characteristics	Assessment Type	No. of Days Assessed	Modality	Participant Assessment Training	Support Tools	Proxy Reporting	Dietary Misreporting
Al-Kutbe, 2017 [19]	Saudi Arabia	Cross- sectional	FL	136	Underweight: 9.5 ± 1.27 Healthy Weight: 9.6 ± 1.1 Overweight: 9.9 ± 1.2 Obese: 9.3 ± 1.2 (8–11)	Primary school-aged girls	FR	4	Written; with follow-up interview	No training described	Visual aids (pictures) to help with portion estimation	Did not use or describe proxy reporting	UR identified by comparisons of EI to BMR; Prevalence: 27 excluded (19.9%)
Andersen, 2014 [20]	Denmark	Cluster-RCT, unblinded, crossover	SB + FL	834	9.98 ± 0.6 (8–11)	School children	FR	7	Self- administered; Web-based Dietary Assessment Software	Children and a parent or guardian received instructions on how to complete the dietary assessment tool	Did not include or describe support tools	Did not use or describe proxy reporting	UR, AR, OR identified by comparisons of EI to BMR Prevalence: UR: 9–14% OR: 2–3%
Arija, 2010 [64]	Spain	Cohort study	FL	258	11.37 ± 0.62 (10–13)	Children at risk of an eating disorder and control participants	FR	3	Written; with follow-up interview (with child)	No training described	Children used photograph album with portion sizes to help during follow-up interview	Child- assisted Proxy Report	Did not use or describe dietary misreporting methods
Bailey, 2018 * [21]	US	Cross- sectional	FL	3235	No mean age provided (0–47.9 months)	Nationally representative sample	24HR	1 (2nd in 23%, 21% and 26% of respondents in 2002, 2008, and 2016, respectively)	Interview administered; used MPM	No training described	Did not include or describe support tools	Single proxy	Did not use or describe dietary misreporting methods
Ball, 2005 [61]	Canada	Longitudinal cohort	FL	135	At risk of overweight: 8.1 ± 0.7 Normal weight: 8.2 ± 0.9 No range provided	Convenience sample of children grades 2–4	FR *Methodology identified as 24HR by authors, but described as a FR*	5	Written; with follow-up interview; used MPM	Participants were provided written instructions and a sample food record	Did not include or describe support tools	Did not use or describe proxy reporting	Did not use or describe dietary misreporting methods
Barkin, 2018 [22]	US	RCT	FL	610	4.3 ± 0.9 (3–5)	Preschool-aged children at risk of obesity	24HR	3	Interview administered	No training described	Did not include or describe support tools	Single proxy	Did not use or describe dietary misreporting methods
Bayindir-Gümus 2020 [23]	Turkey	Cross- sectional	FL	70	26.6 ± 14.8 months (6–60 months)	Patients visiting well-child clinics as outpatients	FR	3	Written	No training described	Food and nutrition photograph catalog	Single proxy	Did not use or describe dietary misreporting methods
Bernabe- García, 2019 [24]	Mexico	Cross- sectional	FL	101 *N* = 20 preschoolers *N* = 64 school-age *N* = 17 adolescents	Preschoolers: Median: 5 (3.12–5.9) School-age: Median: 8.5 (6–11.7) Adolescents: Median: 13.7 (12–18.9)	Male patients with Duchenne Muscular Dystrophy	24HR	3	Interview administered; used MPM	No training described	Food models	Unclear—describes patients and parents reporting, but did not provide additional details	Did not use or describe dietary misreporting methods
Börnhorst, 2014 [12]	Sweden, Germany, Hungary, Italy, Cyprus, Spain, Belgium, Estonia	Cross- sectional	FL, SB	9560	No mean age provided (2–9)	European children	24HR	1 (2nd in ~26% of original sample)	Computerized, self- administered children and infants nutrition assessment	No training described	Standardized photographs displayed on screen to help with portion estimation	Multiple-proxy approach: Parents completed, then teachers and school staff were interviewed	Black [70] and Goldberg [71] cutoffs Prevalence: UR: *n* = 1150 (9.5%) OR: *n* = 407 (3.4%)
Campbell, 2006 [51]	Australia	Cross- sectional	FL	560	6.1 (5–6)	Children (no other specific characteristics)	FFQ 56-items; assessed intake over past week	N/A	Written	No training described	Did not include or describe support tools	Single proxy	Plausibility cutoffs (>17,601 kJ) Prevalence: OR: *n* = 7
Coufopoulos, 2003 [62]	UK	Longitudinal study	FL	105	No mean age provided (11–13)	Children (no other specific characteristics)	FR	3	Written, with follow-up interview	No training described	Food photograph atlas used in follow-up interview	Did not use or describe proxy reporting	Comparisons of EI and BMR ratios Prevalence: UR: *n* = 44 (42%)
da Silva, 2021 [25]	Brazil	Cross- sectional	FL	797	Median: 9.7 (9.5–10)	Representative sample of children in 4th grade	24HR	2	Interview administered via telephone;	No training described	Household measurement tools (i.e., glasses, spoons)	Did not use or describe proxy reporting	Plausibility cutoffs (<2092 kJ or >20,920 kJ) Prevalence: UR: *n* = 2 OR: *n* = 16
Davison, 2013 [26]	US	Pre–post cohort design	FL	154	3.59 ± 1.01 (2–5)	Preschool-aged children enrolled in Head Start	24HR	2	Interview administered via telephone; used MPM	No training described	Did not include or describe support tools	Single proxy	Did not use or describe dietary misreporting methods
Deierlein, 2014 [27]	US	Cross- sectional	FL	1010	No mean age provided (6–8)	Children (no other specific characteristics)	24HR	4	Interview administered	No training described	Did not include or describe support tools	Single proxy	Plausibility cutoffs (<2092 kJ or >20,920 kJ) Prevalence: UR: *n* = 23 OR: *n* = 1
Elbel, 2015 [28]	US	Difference-in-difference	FL	2172 (FFQ) 363 (FFQ + 24HR)	No mean age provided (3–10)	Children living in South Bronx	24HR + FFQ FFQ: Did not give information on number of items or assessment timeframe	1 (24HR)	Interview administered	No training described	FFQ: Used 4 oz serving cups as reference during the survey	Single proxy	Did not use or describe dietary misreporting methods
Eldridge, 2019 * [29]	US	Cross- sectional	FL	9473 FITS 2002: 2962 FITS 2008: 3276 FITS 2016: 3235	No mean age provided (6–47.9 months)	Nationally representative sample	24HR	1 (2nd in 23%, 21% and 26% of respondents in 2002, 2008, and 2016, respectively)	Interview administered; used MPM	No training described	Measuring cups and tape; list of commonly forgotten foods; visual aid booklet	Single proxy	Did not use or describe dietary misreporting methods
Elliott, 2011 [52]	Australia	Cross- sectional	FL	2460	Grade 1: Male: 6.2 ± 0.36 Female: 5.1 ± 0.38 Grade 5: Male: 10.2 ± 0.4 Female: 10.1 ± 0.4 Grade 10: Male: 15.2 ± 0.4 Female: 15.2 ± 0.4 (5–17)	Nationally representative sample	FR (completed by all children in grades 5 and 10, and 25% in grade 1)	1	Written with follow-up interview	Children were shown how to complete the food record during classroom presentation	Measuring cups, spoons, rulers	Did not use or describe proxy reporting	McCrory et al. [72] cutoffs using ratio of reported EI to predicted TEE. Prevalence: 45% of original sample had implausible EI
Epstein, 2002 [66]	US	Cross- sectional	FL	13	10.4 ± 1.7 (8–12)	Children without obesity	24HR	3	Interview administered	No training described	Measuring cups, ruler	Child- assisted proxy report	Did not use or describe dietary misreporting methods
Epstein, 2005 [67]	US	Within- subject design	FL	13	11.2 ± 1.1 (8–12)	Children with overweight (at or above 85th percentile)	24HR	3	Computerized; used MPM	Families were trained on how to complete measures prior to baseline session	Food models, measuring cups and spoons, ruler	Did not use or describe proxy reporting	Did not use or describe dietary misreporting methods
Epstein, 2021 [30]	US	Cross- sectional	FL	158	7.69 ± 1.15 (6–9)	Children (no other specific characteristics)	FFQ Block Kids FFQ (2004) Quantitative; 77 items; assessed consumption over a 1-week period	N/A	Written	No training described	Food images to help with portion estimation	Single proxy	Institute of Medicine Approach (compared EI to estimated EER needed to maintain body weight); UR = <70% of this value Prevalence: UR: 39% of original sample
Evans, 2016 [31]	England	Cross- sectional	FL + SB	2709	7.02 ± 0.3 (6–8)	Primary school children	FR (Child and Diet Evaluation Tool)	1	Written	No training described	Did not include or describe support tools	Multiple-proxy approach: during the day, trained administrators completed records; after school, parents completed	Plausibility cutoffs (above 4000 kcal/day) Prevalence: OR: *n* = 37 (1.4%)
Hart, 2022 [32]	US	RCT	FL	78	9.6 ± 1.0 (8–11)	Healthy children reporting average time in bed ≤ 9.5 hrs/night	24HR	3	Interview administered via telephone; used MPM	Instructions were provided to participants	Aids for portion size estimation (did not provide more details)	Did not use or describe proxy reporting	Did not use or describe dietary misreporting methods
Hassapidou, 2009 [53]	Greece	Cross- sectional	FL	276	9.98 ± 0.95 (8–12)	Children (no other specific characteristics)	24HR and FFQ FFQ: Did not describe number of items/assessment timeframe	Did not describe	Did not describe	No training described	Did not include or describe support tools	Did not use or describe proxy reporting	Did not use or describe dietary misreporting methods
Hendrie, 2011 [65]	Australia	Cluster RCT	FL	145	8.6 ± 2.96 (4–13)	Healthy children who were regular fat dairy consumers	24HR	3	Interview administered; used a 3-pass approach	No training described	Food model booklet	Child- assisted proxy report for children (<10 yrs)	Did not use or describe dietary misreporting methods
Ip, 2018 [33]	US	Longitudinal cohort study	FL	237	No mean age provided (2.5–3.5)	Latino children from farm worker families	24HR	3	Interview administered; used MPM	No training described	Serving size guide; interviewer directly measured bowl and items in the family’s home	Multiple proxy approach: For children attending preschool, data were collected from those individuals directly with the mothers’ permission	Did not use or describe dietary misreporting methods
Jamshidnia, 2021 [34]	Iran	Case–control	FL	342	Children with ADHD: 8.7 ± 1.67 Controls: 8.7 ± 1.63 (7–13)	Children with ADHD and matched controls	FFQ 186-items; assessed intake over daily, weekly, or monthly period depending on food group	N/A	Interview administered	No training described	Food photograph booklet	Child- assisted proxy report	Excluded UR/OR, but did not say how this was determined Prevalence: *n* = 18
Jildeh, 2011 [54]	Palestine	Cross- sectional	FL	313	No mean age provided (11–16)	School-aged children	24HR	1	Interview administered	No training described	Food intake booklet	Did not use or describe proxy reporting	Did not use or describe dietary misreporting methods
Kolahdooz, 2016 [35]	US	Cross- sectional	FL	84	5–8: Boys: 7.1 ± 1.2 Girls: 7 ± 1.1 9–13: Boys: 11 ± 1.5 Girls: 11 ± 1.5 14–16: Boys: 15 ± 0.9 Girls: 16 ± 0.9 (5–16)	Urban African Americans with low income	24HR	1	Interview administered, face-to-face	No training described	Household utensils, food models of commonly consumed items; forgotten foods list	Single proxy for children <8 years	Plausibility cutoffs: Excluded EI > ±2 SD from the mean intake *n* = 3 excluded
Kral, 2016 [36]	US	Crossover design with repeated measures	LB + FL	40	9.4 ± 0.8 (8–10)	Children (no other specific characteristics)	FR	1 (repeated 3 times for each condition)	Written	Caregivers received detailed training, were given written instructions, and provided an example of a completed FR	Visual aids	Single proxy	Did not use or describe dietary misreporting methods
Labadarios, 2005 [55]	South Africa	Cross- sectional	FL	2894	No mean age provided (1–9)	Nationally representative sample	24HR + FFQ FFQ: Assessed intake over past 6 months for children ≥2 years; assessed intake over 1 month for children 12–23 months	1 (24HR)	Interview administered	No training described	Food models, household utensils and empty containers, samples of dry foods presented	Single proxy	Did not use or describe dietary misreporting methods
Lahoz-García, 2015 [37]	Spain	Cross- sectional	FL	320	10 ± 0.5 (9–11)	4th and 5th grade primary school children	24HR	2	Computerized, self-administered Young Adolescents Nutrition Assessment on Computer (YANA-C)	No training described	Did not include or describe support tools	Did not use or describe proxy reporting	Did not use or describe dietary misreporting methods
Lee, 2020 [38]	Canada	Within- subject repeated measure	FL, LB	21	11.7 ± 0.4 (9–14)	Children with normal or overweight	FR	1 (repeated 5 times for each study day)	Written	No training described	Did not include or describe support tools	Single proxy	Did not use or describe dietary misreporting methods
Li, 2017 [39]	US	RCT	FL	22 7–12: *n* = 12 13–18: *n* = 10	Median age: 11.5 (7–18)	Survivors of pediatric acute lymphocytic leukemia	FR *Methodology* *identified as 24HR by* *the* *authors, but* *described as a FR*	3	Written	Participants were given instructions on how to complete a FR	Did not include or describe support tools	Unclear (mentions completed by parents or children, but does not specify when or how)	Did not use or describe dietary misreporting methods
Lioret, 2009 [56]	France	Cross-sectional	FL	1030 3–6 yrs: *n* = 244 7–10 yrs: *n* = 330 11–14 yrs: *n* = 456	No mean age provided (3–14)	Nationally representative sample	FR	7	Written with follow-up interview	Caregivers/ children completed a 45–60 min training on how to complete the FR and other measures	Food photograph booklet	Child- assisted proxy report (children <10 years)	Plausibility cutoffs: Excluded if log- transformed value of EI was < mean-3 SD within the age groups Prevalence: UR: *n* = 5
López-Frías, 2005 [57]	Spain	Cross- sectional	FL + SB	521	No mean age provided (6–11)	Nationally representative sample	FR + FFQ FFQ: Did not describe number of items or assessment timeframe	4 (FR)	Written	Participants were given instructions on how to complete the assessments	Did not include or describe support tools	Multiple proxy approach: School canteen provided menu, ingredients and recipes for items offered; trained staff weighed foods at school	Did not use or describe dietary misreporting methods
Luo, 2021 [40]	US	Cross- sectional	FL	159	8.5 ± 0.96 (7–11)	Children with exposure to gestational diabetes or maternal obesity in utero	24HR	Average of 2 per person	Interview administered face-to-face; used MPM	No training described	Did not include or describe support tools	Did not use or describe proxy reporting	Plausibility cutoffs: Excluded recalls exceeding 3 SD of expected mean value of regression of body weight compared to EI. Prevalence: *N* = 2 excluded
Magkos, 2006 [58]	Greece	Cross- sectional	FL	198	11.6 ± 0.4 (10.8–13.5)	School children in rural Greece	24HR	1	Interview administered; used MPM	No training described	Household measures (cups, spoons); picture food models	Did not use or describe proxy reporting	Did not use or describe dietary misreporting methods
Morais, 2024 [41]	São Tomé and Príncipe	Cross- sectional	FL	546	10.82 ± 1.17 (9–16)	Children and adolescents in Cantagalo, São Tomé, and Príncipe	24HR	1	Interview administered	No training described	Visual aids; direct reading of food labels	Did not use or describe proxy reporting	Goldberg method [71] Prevalence: Boys: UR: *n* = 63; OR: *n* = 4 Girls: UR: *n* = 45; OR: *n* = 5
Overcash, 2019 [42]	US	Non- randomized cluster- allocated community trial	FL	103	No mean age provided (9–12)	Children from households with low income	24HR	3	Interview administered face-to-face	No training described	Household utensils (cups, bowls, etc.); food models; food amounts booklet	Did not use or describe proxy reporting	Plausibility cutoffs: <500 kcal/day or >7 cups of vegetables reported Prevalence: *N* = 29 (individual recall days excluded)
Reed, 2013 [43]	US	Cross- sectional	FL	43	11 ± 0.93 (10–12)	Girls residing in low- income African American communities	FFQ (revised Block Kids 2004 FFQ) 72 items; assessed over a 1-week period	N/A	Written or interview administered (given option)	No training described	Pictures embedded in FFQ to help with estimating portions	Did not use or describe proxy reporting	Did not use or describe dietary misreporting methods
Roche, 2007 [59]	Peru	Cross- sectional	FL	36 3–4 yrs: *n* = 19 4–6 yrs: *n* = 17	No mean child age provided (3–6)	Awajún children	24HR	2	Interview administered face-to-face	No training described	Local foods used as references; household utensils (cups, plates, bowls); used food scale for direct weighing of local foods	Single proxy	Did not use or describe dietary misreporting methods
Rohde, 2017 [44]	Denmark	RCT	FL	285	Intervention group: Median: 4.0 Control group: Median: 3.9 (2–6)	Danish preschool children w/high birth weight, high maternal pre- pregnancy BMI or low maternal education	FR	4	Written	No training described	Picture booklet with portion sizes	Single proxy	Did not use or describe dietary misreporting methods
Ruel, 2006 [68]	Mexico	Cross- sectional beneficiary/matched control survey	FL	518	Beneficiary children: 3.5 ± 0.9 Control children: 3.7 ± 0.9 (2–5)	Beneficiary children selected from hogares in Guatemala City and matched case controls	Direct weighing (foods in hogares) Recall approach for foods consumed outside of direct weighing period 48 h recall approach for weekend days	3 (1 weekday, 2 weekends)	Did not describe	No training described	Did not include or describe support tools	Multiple proxy approach: Direct weighing of foods consumed in hogares; parent/caregiver recall of foods consumed outside of hogares	Did not use or describe dietary misreporting methods
Sadik, 2010 [69]	Ghana	Non- experimental, descriptive action research	FL	40 1–3 yrs: *n* = 5 7–10 yrs: *n* = 7 11–14 yrs: *n* = 17 15–18 yrs: *n* = 11	No mean age provided (1–14)	Children living in orphanages in Ghana	FFQ Quantitative: Did not describe number of items or assessment timeframe	N/A	Interview administered	No training described	Food models	Single proxy (for children <5 years)	Did not use or describe dietary misreporting methods
Saelens, 2018 [45]	US	Prospective observational study	FL	618	Group 1: Low PAE/Low NE: 9.1 ± 1.5 Group 2: Low PAE/High NE: 9.0 ± 1.6 Group 3: High PAE/Low NE: 9.0 ± 1.6 Group 4: High PAE/High NE: 9.3 ± 1.5 (6–12)	Children residing in King County, Washington, and San Diego County, California	24HR	3	Interview administered via telephone; used MPM	No training described	2D food models	Consensus recall approach for children ≤ 8 years	Did not use or describe dietary misreporting methods
Serra-Majem, 2001 [60]	Spain	Cross- sectional	FL	3534 2–5 yrs: *n* = 385 6–9 yrs: *n* = 423 10–13 yrs: *n* = 567 14–17 yrs: *n* = 682 18–24 yrs: *n* = 1477	No mean age provided (2–24 years)	Nationally representative sample of school-children and adolescents	24HR + FFQ FFQ: Quantitative; 176 items; did not describe assessment timeframe	24HR: 1 in full sample, repeated in 25–30% of sample	Interview administered	No training described	Measured household items	Multiple proxy approach for children ≤ 5 years: Completed by mothers or caretaker, then additional information obtained via telephone interviews with school food service directors as needed	UR identified by comparison of EI to predicted BMR Prevalence: Did not describe
Sharkey, 2012 [46]	US	Cross- sectional	FL	50	9.1 ± 1.3 (6–11)	Mexican-origin children	24HR	3	Interview administered; used modified MPM approach	No training described	Measured typically used glassware, cups, containers in home; food and beverage models; geometric shapes; 3-dimensional thickness aids	Did not use or describe proxy reporting	Did not use or describe dietary misreporting methods
Shiau, 2017 [47]	South Africa	Cross- sectional	FL	440	6.7 ± 1.4 (5–9)	Children w/HIV (matched with controls)	24HR + FFQ FFQ: Quantitative; did not describe number of items; assessed intake over past 6 months	1 (24HR)	Interview administered	No training described	Food flash cards; food photo manual	Child- assisted proxy report	Did not use or describe dietary misreporting methods
Sonneville, 2008 [63]	US	Prospective observational study	FL	538	11.7 ± 0.75 No range provided	School-children (enrolled in grades 6 and 7)	FFQ Semi-quantitative; did not describe number of items or assessment timeframe	N/A	Did not describe	No training described	Did not include or describe support tools	Did not use or describe proxy reporting	Plausibility cutoffs: <500 kcal/day or >7000 kcal/day Prevalence: Did not describe number excluded for misreporting
Yang, 2017 [48]	Malaysia	Cross- sectional	FL	236	9.9 (Confidence interval: 9.8–10.0) (8–12)	Primary school children	24HR + FFQ FFQ: 13-item food habits questionnaire; assessed intake over past 6 months	2 (24HR)	Interview administered; used MPM	No training described	Food photographs; local household measures; direct reading of food packaging	Did not use or describe proxy reporting	Black and Cole Method [70] Prevalence: 95/236 (~40%) identified as misreports
Zakarneh, 2023 [49]	Jordan	Cross-sectional	CB	107 6–8 yrs: *n* = 22 9–13 yrs: *n* = 51 14–18 yrs: *n* = 34	HbA1c < 8.5 (*n* = 36) 11.9 ± 3.2 HbA1c > 8.5 (*n* = 71) 12 ± 3.4 (6–18)	Children (6–18 yrs) diagnosed with type 1 diabetes between ages 1–10; acute care at time of data collection	FFQ 120-items; validated; assessed intake over previous 12 months	N/A	Interview administered	No training described	Food models	Single proxy	Did not use or describe dietary misreporting methods
Zeković, 2022 [50]	Republic of Serbia	Cross- sectional	FL	576 1–3 yrs: *n* = 290 3–9 yrs: *n* = 276	Toddlers (1–3 years): 1.98 ± 0.64 Children (3–9) 6.01 ± 1.87 (1–9)	Nationally representative sample	FR	2	Written; followed by face-to-face interview with caregiver to confirm details	No training described	Photographic food atlas	Single proxy	Did not use or describe dietary misreporting methods

Notes. * Bailey et al. [21] and Eldridge et al. [29] used the same Feeding Infants and Toddlers (FITS) 2016 dataset in the study sample. Abbreviations: ADHD: Attention-deficit/hyperactivity disorder; AR: Acceptable report; BMR: Basal metabolic rate; CB: Clinic-based; EER: Estimated energy requirements; EI: Energy intake; FFQ: Food frequency questionnaire; FL: Free-living; FR: Food record; HbA1c: Hemoglobin A1C; HIV: Human immunodeficiency virus; kJ: kilojoule; LB: Lab-based; MPM: Multiple-pass method; NE: Nutrition environment; OR: Over-report; PAE: Physical activity environment; SB: School-based; SD: Standard deviation; TEE: Total energy expenditure; UR: Under-report; 24HR: 24 h dietary recall.

Details on assessment administration showed that for 24HR, the total number of days assessed ranged from a single administration to 4 days, with more studies using multiple days (*n* = 17) [22,24,25,26,27,32,33,37,40,42,45,46,48,59,65,66,67] than single administrations (*n* = 11) [12,21,28,29,35,41,47,54,55,58,60]. One study did not describe the number of days assessed [53]. A multiple-pass method was described in thirteen studies using a 24HR [21,24,26,29,32,33,40,45,46,48,58,65,67]. For FR, the total number of days assessed ranged from one day [31,36,38,52] to seven days [20,56]. Two studies described implementation of a multiple-pass approach when using a FR [50,61]. For FFQ assessments, five were described as semi-quantitative [28,30,43,49,51], and five were described as quantitative [34,47,55,60,69]. Other studies that used FFQ did not provide enough detail to determine the type used (*n* = 3) [53,57,63]. The timeframe of recall for FFQs varied, with some assessing usual intake over the previous week [30,43,51], 6 months [47,48,55], or 12 months [49]. Total items ranged from 13 [48] to 186 [34]. The Block Kids FFQ was used in two studies [30,43]; however, no other studies used the same FFQ for assessment. Several studies provided little information on the specific FFQ, including name, time frame, and/or number of items [28,53,55,57,63,65,69].

The modality of assessment was described in all but two studies [53,68]. For 24HR, most studies (*n* = 25) used interview administrations [21,22,24,25,26,27,28,29,32,33,35,40,41,42,45,46,47,48,54,55,58,59,60,65,66], with fewer using computerized modalities (*n* = 3) [12,37,67]. Computerized modalities were often used in children over 8 years [37,67] with only one study using this modality in children younger than 8 years [12]. For FFQ, interview administration was most frequently used (*n* = 8) [28,34,47,48,49,55,60,69], followed by written (*n* = 3) [30,51,57]. One study by Reed et al. [43] provided participants the option to have written or interview administration, and two studies did not describe modality [53,63].

Only nine studies (17%) reported providing training on the dietary assessment for caregivers and children [20,32,36,39,52,56,57,61,67]. Seven of these used FR for dietary assessment [20,36,39,52,56,57,61], and two used a 24HR [32,67]. Examples of training included providing written and/or verbal instructions on how to complete the dietary assessment tool (*n* = 4) [20,36,39,61], completing in-person training on how to complete the measures (*n* = 2) [52,56], and providing example food records (*n* = 2) [36,61]. Several studies (*n* = 3) acknowledge that caregivers and children were trained, but did not provide additional details on how or what they were trained on related to the dietary assessment [32,57,67]. For all studies reporting on training for caregivers and children, not enough detail was provided to determine specific training related to support tools or proxy reporting.

### 3.4. Support Tools for Dietary Assessment

More than two-thirds of studies (*n* = 36, 69%) used support tools for dietary assessment [12,19,23,24,25,28,29,30,32,33,34,35,36,41,42,43,44,45,46,47,48,49,50,52,54,55,56,58,59,60,62,64,65,66,67,69]. Across all age ranges, the most utilized supports included visual aids (i.e., picture reference guides, food amounts booklets, etc.) to assist with portion size estimation (*n* = 20) [12,19,23,29,30,32,33,34,36,41,42,43,44,46,47,50,54,56,62,64], food models (*n* = 11) [24,35,42,45,46,49,55,58,65,67,69], and providing standardized household utensils (i.e., measuring cups, spoons, bowls) (*n* = 10) [25,28,29,35,42,52,55,58,66,67]. Less commonly used supports included using or measuring household items found in participants’ homes or communities (*n* = 5) [33,46,48,59,60], rulers (*n* = 3) [52,66,67], direct reading of food packaging or weighing items (*n* = 3) [41,48,59], having samples of foods available during the assessment (*n* = 2) [55,59], forgotten food lists (*n* = 2) [29,35], and food photographs, the only technology-based support tool (*n* = 1) [48]. When support tools were used, most studies used a single tool to support dietary assessment (*n* = 23, 64%) [12,19,23,24,25,28,30,32,34,36,43,44,45,47,49,50,54,56,60,62,64,65,69], with fewer studies (*n* = 13, 36%) using multiple supports [29,33,35,41,42,46,48,52,55,58,59,66,67].

Supports utilized varied across life stages (Table 4). Of the seven studies conducting dietary assessment only among children ≤ 5 years, three (43%) included and/or described using support tools [23,29,33]. Four different supports were used across studies; however, visual aids were the most prevalent [23,29,33]. One study used visual aids as the sole support tool [23], whereas two studies described using multiple supports during the assessment [29,33]. Eldridge et al. [29] combined visual aids, providing standardized household items and a frequently forgotten food list, whereas Ip et al. [33] combined visual aids with measuring the size of bowls and dishes in the family’s home.

Twelve studies (60%) conducting dietary assessment among children only between the ages of 6 and 12 years used support tools [19,25,30,32,36,42,43,45,46,48,66,67]. Seven different supports were described; the most common were visual aids (*n* = 7) [19,30,32,36,42,43,46], followed by providing standardized household items (*n* = 4) [25,42,66,67] and food models (*n* = 4) [42,45,46,67]. Additional tools, such as using or measuring household items (*n* = 2) [46,48], rulers (*n* = 2) [66,67], direct reading of food packaging (*n* = 1) [48], and food photographs (*n* = 1) [48] were used, but less consistently across the age group. Of the 12 studies using support tools, seven described using a single support tool [19,25,30,32,36,43,45]. Only five (42%) described using multiple supports [42,46,48,66,67]; however, the combination of supports varied across studies.

Among studies with samples of children 6 years and older, seven (70%) used additional tools to support dietary assessment accuracy [34,41,49,54,58,62,64]. Across these studies, four different supports were used, most commonly visual aids (*n* = 5) [34,41,54,62,64], followed by food models (*n* = 2) [49,58], provision of standardized household items (*n* = 1) [58], and direct reading of food labels (*n* = 1) [41]. Five studies described the use of a single support tool [34,49,54,62,64], whereas two studies described the use of multiple support tools [41,58]. However, the combination of support differed by study; Magkos et al. [58] combined food models and provided standardized household items, whereas Morais et al. [41] combined visual aids and direct reading of food labels.

Support tools were used most consistently among mixed-age samples, with fourteen studies (93%) utilizing them for dietary assessment [12,24,28,35,44,47,50,52,55,56,59,60,65,69]. Eight different support tools were used, and visual aids (*n* = 5) [12,44,47,50,56] and food models (*n* = 5) [24,35,55,65,69] were the most common, followed by provision of standardized household items (*n* = 4) [28,35,52,55]. Other supports, including using or measuring household items (*n* = 2) [59,60], displaying samples of food items (*n* = 2) [55,59], forgotten food lists (*n* = 1) [35], rulers (*n* = 1) [52], and direct weighing of foods (*n* = 1) [59], were used less frequently. Ten studies described using a single support for dietary assessment [12,24,28,44,47,50,56,60,65,69], whereas four studies used multiple supports, and all provided standardized items; however, the additional support varied among the studies [35,52,55,59].

### 3.5. Proxy Reporting

Thirty-one studies (60%) described using proxy reporting for dietary assessment, and it was used across all types of self-reported assessment (FFQ, FR, and 24HR) [12,21,22,23,26,27,28,29,30,31,33,34,35,36,38,44,45,47,49,50,51,55,56,57,59,60,64,65,66,68,69]. Across all ages, most studies used single proxies (*n* = 18) [21,22,23,26,27,28,29,30,35,36,38,44,49,50,51,55,59,69]. Other types of proxy reporting, including child-assisted reports (*n* = 6) [34,47,56,64,65,66], multiple-proxy approaches (*n* = 6) [12,31,33,57,60,68], and a consensus recall approach (*n* = 1) [45], were utilized less consistently. In two studies, it was unclear whether proxy reporting was used; assessments were completed by either parents or children without specifying the primary reporter [24,39].

Table 5 summarizes the use of proxy-reporting types across life stages. Proxy reporting was described in all studies that conducted dietary assessment in children only aged ≤ 5 years [21,22,23,26,29,33,68]. Two studies by Sadik et al. [69] and Serra Majem et al. [60] included participants with wider age ranges, but specified that proxy reporting was used for only children ≤ 5 years. Single-proxy reporting was the most common method used (*n* = 6) [21,22,23,26,29,69] followed by multiple-proxy approaches (*n* = 3) [33,60,68]. The implementation of multiple-proxy approaches varied across studies. Ip et al. [33] conducted a 24HR with the primary caregiver, but in cases when the child attended childcare, the research staff asked for permission to interview childcare workers directly about consumption. Ruel et al. [68] utilized a direct observation approach for foods consumed while at childcare, then a recall with the caregivers for foods consumed outside of childcare settings. Finally, Serra-Majem et al. [60] described that when needed, telephone interviews were conducted with food service directors to gather information on foods offered in childcare or school settings.

Seven studies (35%) described proxy-reporting methods for study samples of children aged 6–12 years only [27,30,31,36,45,57,66]. More variability was observed in the type of proxy reporting described, including single-proxy reporting (*n* = 3) [27,30,36], multiple-proxy approaches (*n* = 2) [31,57], child-assisted proxy reporting (*n* = 1) [66], and a consensus recall approach (*n* = 1) [45]. Child-assisted proxy reporting included parents or caregivers providing the initial report, followed by confirming details with the child. Implementation differed across multiple-proxy approaches. Using a FR, Evans et al. [31] trained administrators to complete records during school hours, followed by parent recording for meals consumed outside of school hours. Lopez-Frias et al. [57] also used an FR method and described trained staff being present for meals consumed at school, as well as obtaining menu and ingredients directly from school food service workers; however, no description was provided regarding whether the parent or child was responsible for completing reporting for other eating occasions.

Proxy reporting was described in four studies (40%) with samples of children ≥ 6 years. Single proxies (*n* = 2) [38,49] and child-assisted proxy reporting (*n* = 2) were described [34,64]. Eighty-five percent (*n* = 11) of studies conducted among mixed-age samples described utilizing proxy-reporting methods [12,28,35,44,47,50,51,55,56,59,65]. Single-proxy reporting was the most prevalent method described (*n* = 7) [28,35,44,50,51,55,59] followed by child-assisted proxy reporting (*n* = 3) [47,56,65], and a multiple-proxy approach (*n* = 1) [12]. Bornhorst et al. [12] described a multiple-proxy approach involving direct observation of meals consumed at school and interviews with teachers and food service staff to supplement information obtained from primary proxies. Across all studies using proxy reporting, few details about the proxy reporters were included. Most studies described “parents or caregivers” or “mothers” as proxies but did not provide additional details, such as age, race or ethnicity, or other sociodemographic factors that might lead to variability in completing the proxy role (e.g., education level, parity, etc.).

### 3.6. Dietary Misreporting

#### 3.6.1. Dietary Misreporting Methods

Nineteen studies (37%) described specific methods used to identify dietary misreporting [12,19,20,25,27,30,31,34,35,40,41,42,48,51,52,56,60,62,63]. The most prevalent method across all age ranges included plausibility cutoffs for EI *(n* = 9) [25,27,31,35,40,42,51,56,63]. These cutoffs included predefined EI ranges (i.e., excluding reports <500 kcal or >5000 kcal/day) [25,27,31,42,63] or excluded reports of EI falling outside of a specified number of standard deviations from the mean intake [35,40,51,56]. Four studies used minimum energy consumption of <500 kilocalories per day (kcal/day) for implausible under-reports (UR) [25,27,42,63]. For studies using maximum energy cutoffs to indicate implausible over-reports (OR) of EI, cutoffs varied and included greater than 4000 [31], 5000 [25,27], or 7000 calories [63]. Overcash et al. [42] used a threshold of reporting >7 cups of vegetables per day to indicate implausible reports.

Additional methods included comparisons of EI to basal metabolic rate (BMR) (*n* = 4) [19,20,60,62], estimated total energy expenditure (TEE) (*n* = 1) [52], and estimated energy requirements (EER) (*n* = 1) [30]. Three studies specifically mention using the Black and/or Goldberg criteria, which involves a comparison of the ratio of EI to BMR, while also accounting for physical activity level [12,41,48]. One study acknowledged that implausible intakes were excluded, but did not provide additional details regarding how these were identified [34]. Methods for determining dietary misreporting were used across all assessment types (FFQ, FR, and 24HR). However, almost all studies that described these methods (*n* = 17, 89%) were observational study designs, and most frequently occurred using cross-sectional designs (*n* = 14) [12,19,25,27,30,31,34,35,40,41,48,51,52,56,60,62,63]. Accounting for dietary misreporting was less common among experimental designs (*n* = 2) where a diet behavior was manipulated, and changes in energy intake were an outcome [20,42].

When comparing methods to detect dietary misreporting across age ranges (Table 6), studies that assessed dietary intake only in children ≤ 5 years did not use methods to determine dietary misreporting. A variety of methods were used to detect dietary misreporting among studies assessing intake among children 6 to 12 years *(n* = 9; 45%) [19,20,25,27,30,31,40,42,48]. The most prevalent method included plausibility cutoffs (*n* = 5) [25,27,31,40,42]. Two studies by da Silva et al. [25] and Deierlein et al. [27] used the same cutoffs [<2092 kJ (<500 kcal/d) or >20,902 kJ (>5000 kcal/d)]. However, the remaining studies used variable cutoffs to determine implausible UR or OR. Fewer studies used comparisons of reported EI to BMR, EER, or TEE (*n* = 2) [19,30] or the Black Criteria (*n* = 2) [20,48].

For studies including children 6 years and older, four studies (40%) used methods to detect dietary misreporting [34,41,62,63]. Each study used different methods, including plausibility cutoffs (<500 kcal/day or >7000 kcal/day) [63], comparisons of EI and BMR [62], and the Goldberg criteria [41]. One study did not provide enough detail to determine how dietary misreporting was captured [34]. Six studies (40%) with mixed-age samples used methods to detect dietary misreporting [12,35,51,52,56,60]. Plausibility cutoffs were the most used method (*n* = 3) [35,56,73], with all studies excluding reports of EI that fell outside a specified number of standard deviations from the mean intake. However, the specific thresholds varied across studies, ranging from 2 to 3 standard deviations. Additional methods include the Black and Goldberg criteria [12], comparison of reported EI to predicted TEE [52], and comparison of reported EI to BMR [60].

#### 3.6.2. Prevalences of Dietary Misreporting

Across all age groups, prevalence of dietary misreporting was described in 17 (89%) studies that employed methods to detect dietary misreporting [12,19,20,25,27,30,31,34,35,40,41,42,48,51,52,56,62]. Across all 17 studies reporting on this characteristic, some degree of misreporting was captured. However, the way these results were reported varied across studies, with six studies indicating the specific number or percentage of records excluded [34,35,42,48,52,62] and 11 studies describing UR and/or OR [12,19,20,25,27,30,31,40,41,51,56].

For studies included children between the ages of 6 and 12 years only; all studies (*n* = 9) reported on prevalences [19,20,25,27,30,31,40,42,48]. Two studies excluded over one-third of respondents for implausible reports [30,48]. Using comparisons of EI and EER, Epstein et al. [30] excluded 39% of the original sample, whereas Yang et al. [48] used the Black criteria and excluded 95 out of 236 participants (~40%) for implausible intakes. Three studies reported prevalence of both under- and over-reporting [20,25,27]. Andersen et al. [20] and Deierlein et al. [27] found greater prevalences of under-reporting than over-reporting in children ages 8–11 years and 6–8 years, respectively. Among samples of children ≥6 years, the prevalence of misreporting was described in three studies [34,41,62]. Using comparisons of EI and BMR ratios, Coufopolous et al. [62] excluded 42% of the original sample for misreporting. A recent study excluded 63 reports from males and 45 from females for under-reporting, and four reports from males and five from females for over-reporting [41]. Five studies using mixed-age samples reported on misreporting prevalences [12,35,51,52,56]. Elliot et al. [52] used the McCrory et al. [72] criteria, comparing reported EI with predicted TEE, and excluded 45% of food records from children between the ages of 5 and 16 years.

## 4. Discussion

This scoping review describes methods used to support the accuracy of self-reported dietary assessment that captures EI among children 2–12 years in investigations published since 2000. There has been a rising interest in improving dietary assessment methodologies among young children, as current methods have limitations. The Academy of Nutrition and Dietetics and the National Institutes of Health have acknowledged the shortcomings of self-reported dietary assessment methods and the need for improved, innovative solutions to obtain more accurate information on young children’s intake [74,75].

This scoping review identified several areas warranting further research. Included studies used and/or described a variety of support tools (e.g., visual aids and food models); however, their use did not appear to differ across age groups. Among those studies using support tools, only 35% used multiple types of support. Additionally, when multiple supports were used, the combinations varied. While support tools are believed to improve the accuracy of self-reported dietary assessment, to our knowledge, no studies have systematically evaluated whether using one or multiple support tools, or a certain tool or combinations of support tools, improves assessment accuracy among children. Future research should evaluate the added value of a support tool and the combinations of support tools relative to the additional participant burden associated with their use on the increasing accuracy of dietary assessment. Additionally, given that no studies included in this review discuss specifically how support tools were implemented, including how caregivers and children were trained to use the tools during the assessment process, future research should clearly describe how these tools are implemented to support replication.

Additionally, while technology is a growing area of support in older children and adults, this review found very little use of technology to support the accuracy of dietary assessment. The literature among older children and adults has explored using technology, including wearable sensors [13,14,76,77] and food photography [15,16,78] to support dietary assessment. In those populations, technology is believed to help capture the number and timing of eating occasions, as well as relative portion sizes and types of foods consumed more accurately [79]. Therefore, future research investigating how to use technology among younger children and with proxy reporting is warranted, and should include examination of feasibility of implementation, potential increased caregiver burden, privacy concerns, and potential costs.

Since more than half of the included studies, and all studies in which only children ≤ 5 years were included, described the use of proxy reporting, understanding how to improve the accuracy of dietary assessment obtained by proxies is important. Proxy reporting was less commonly used in studies of school-aged children (≥6 years) than in studies of young children (≤5 years). Across all age ranges, single proxies were most often used, while methods involving more than one reporter (i.e., child-assisted proxy reporting, consensus recall approaches, and multiple-proxy approaches) were used less frequently, especially among the youngest children. Only six studies included in this review used a multiple-proxy approach; this seems important given that more than half of children in the US attend regular childcare [11] and thus multiple caregivers are likely to be involved in mealtimes across the day. Across those six studies, implementation of multiple-proxy approaches and the types of assessment varied. Therefore, similarly to what was found with support tools, future research should evaluate the added value of proxy reporters and multiple-proxy reporters relative to the additional participant burden associated with these methods on increasing the accuracy of dietary assessment. It is also important to consider how including multiple proxies may lead to inconsistencies in reporting; therefore, these best practices should address how to transfer information efficiently and effectively and determine whether additional support tools are needed to limit discrepancies. Also, identifying whether a specific age or cognitive development level of the child benefits from proxy reporting methodology is important. Additionally, for all types of proxy reporting (i.e., single-proxy reporting, child-assisted proxy reporting, etc.), developing and reporting on specific protocols, such as how proxies and children were trained and support is received, is warranted. And finally, as previous research shows that proxy characteristics (i.e., age, sex, race) may be related to under- and over-reporting of child intake [80], regularly including this information in future research may help ascertain the influence of proxy characteristics on reporting accuracy. Addressing these areas could improve understanding of young children’s dietary intake during a critical life stage.

Procedures for identifying dietary misreporting were described in less than 40% of studies included in this review and in none of the studies that assessed EI only in children ≤ 5 years. When procedures were described, methodologies varied, but misreporting was most commonly determined using plausibility cutoffs. Among studies that described procedures for dietary misreporting and reported on this characteristic, prevalences ranged from 9 to 24.8% and 2 to 23.4% for over- or under-reporters, respectively. The wide variability in under- and over-reporting is consistent with previous research in children and adolescents, which reported ranges of 2–85% for under-reporting and 3–46% for over-reporting, respectively [6]. Because fewer than half of the studies reported methods for detecting dietary misreporting, and because of observed methodological heterogeneity, the patterns identified and conclusions drawn from this review are limited. Ideally, future research that may be identifying methods to support the increasing accuracy of dietary assessment will identify and report prevalences of dietary misreporting to help determine whether dietary intake is being assessed more accurately. Consistently reporting these characteristics may help ascertain whether newly developed and implemented support tools improve accuracy of dietary assessment and may help refine tools over time.

While this was the first scoping review regarding supports for enhancing self-reported dietary assessment in children aged 2 to 12 years, it does have limitations. The heterogeneity of the included studies, in terms of the types of dietary assessment used, study population characteristics, and settings, may limit comparisons across studies. Findings from this study may not be generalizable to study populations or settings that are not represented in the included evidence. Additionally, this review did not synthesize what enhanced dietary assessment accuracy nor provide a critical appraisal of the included studies. Few studies reported on dietary misreporting, and heterogeneity in methods and tools prevented drawing conclusions about which approaches may improve assessment accuracy. While this limits the ability to comment on the strength of the existing evidence, the approach used is consistent with scoping reviews and supported the objective of this scoping review, which was to map the existing body of literature regarding methods used to support accuracy of self-reported dietary intake in children aged 2 to 12 years by describing characteristics of the available evidence and to identify gaps for future research. Another limitation is that the search strategy included terms such as “energy” and “validity, reliability, etc.” and was limited to studies available in English, which may have excluded relevant studies. Additionally, during full-text screening, a second reviewer was only used for a subset of articles (20%), and a single reviewer conducted data extraction, which may have increased the risk of selection and extraction bias. However, measures were implemented to reduce bias throughout the screening and extraction process, which included achieving an a priori agreement rate for the subset of articles during full-text screening and holding regular check-ins and discussions with the review team as needed. While only one reviewer completed data extraction in October 2024 and November 2025, all included articles were reviewed, and extraction details were confirmed in August 2026 during the manuscript revision phase. Finally, to broadly describe the existing methods used to support accuracy of self-reported dietary intake in children aged 2 to 12 years, some included studies containing children younger than 2 years and older than 12 years. To help with interpretation, the number of children within differing age subgroups was reported, descriptive statistics of child ages were summarized when available, and methods used were summarized by life stages (young children [≤5 years], only school-aged children [6–12 years], children aged ≥6 years, and mixed-age samples which included both young children [≤5 years], and school-aged children and above [≥6 years]).

## 5. Conclusions

Self-reported dietary assessment used varying strategies to support assessment accuracy among children. Across all age groups, studies described a variety of support tools (i.e., visual aids, household utensils); however, using one support tool was more common than using multiple supports, and studies rarely used technology-based supports. Proxy reporting was commonly used to support assessment accuracy, especially in samples in which all children were aged ≤5 years. Across all ages, a single-proxy reporter was most commonly used. Multiple-proxy approaches were used less frequently, and implementation varied. When studies used support tools and proxy reporting, they did not specifically describe how these methods were implemented, including the instructions or training participants received. Fewer than half of the studies reported procedures for identifying dietary misreporting, and when they did, methodologies varied. Future research should evaluate whether individual or combined support tools, which may also include using technology-based approaches and proxy reporters (single or multiple), improve dietary assessment accuracy relative to the burden associated with their use. Studies should also describe in detail how support tools and proxy reporting are implemented to facilitate replication. This includes reporting on the training or guidance provided to proxies on completing assessment, communicating with other caregivers in multiple-proxy approaches, and using additional support tools. Finally, studies should consistently identify and report dietary misreporting prevalence to determine whether dietary intake is assessed more accurately when additional methods, such as support tools or proxy reporting, are used.

## Figures and Tables

**Figure 1:**
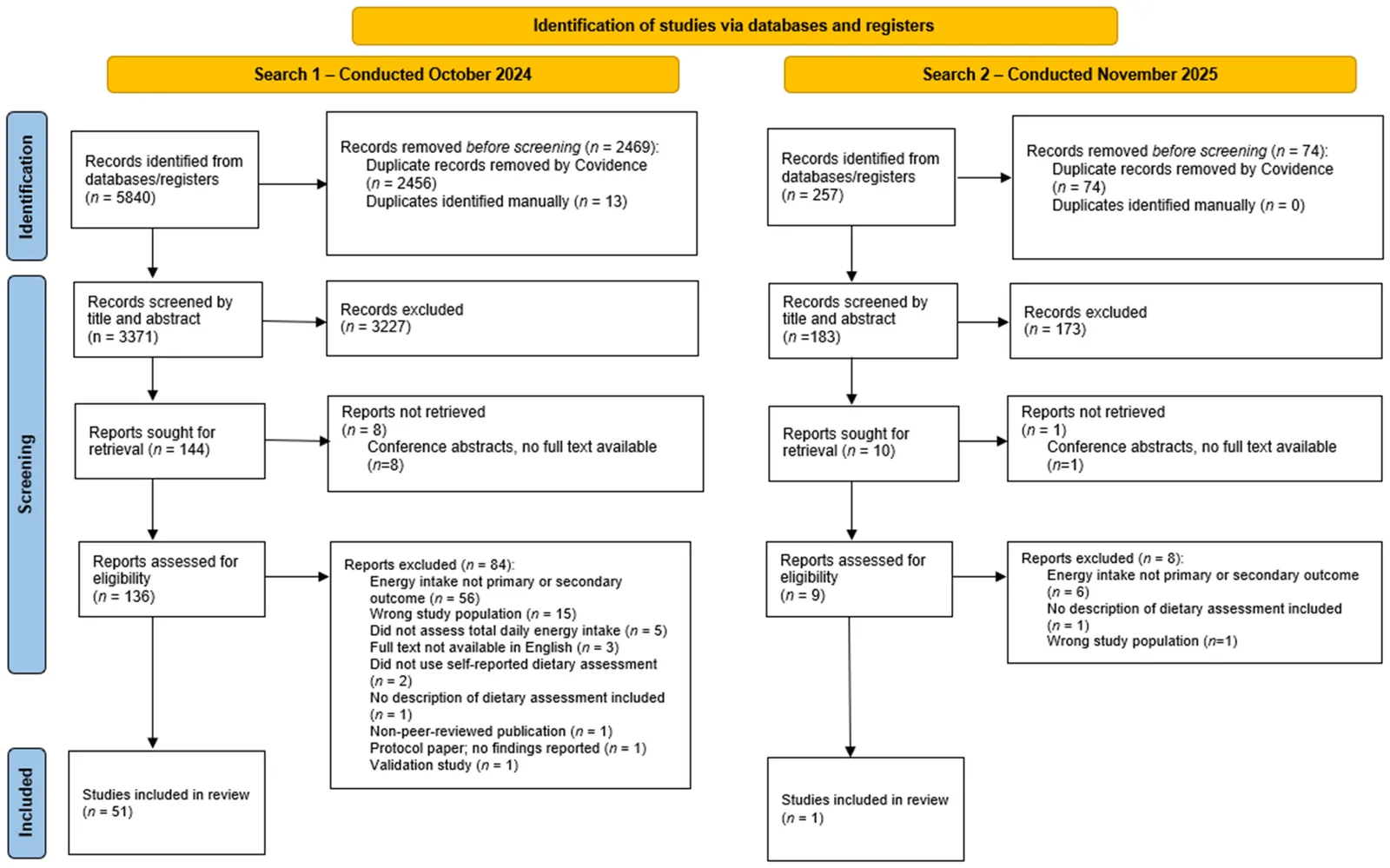
PRISMA flow chart depicting the two literature searches and study selections for self-reported dietary assessment methodologies capable of capturing total energy intake.

**Table 1:** Summary of search strategy by database for search conducted on 13 October 2024 and 18 November 2025.

Database	Search Terms/Strategy
Web of Science (WoS), CINAHL (EBSCO), CABI (WoS), Education Source (EBSCO), and ERIC (EBSCO)	(diet* OR nutrition OR eating* OR food*) AND (assessment* OR evaluation* OR measurement*) AND (energy) AND (child* OR “young person” OR youth* OR “young people”) AND (validity OR validation OR reliable OR reliability OR accuracy)
PubMed (MEDLINE)	(((diet* OR nutrition OR eating* OR food*) AND (assessment* OR evaluation* OR measurement*) AND (energy)) AND (child* OR “young person” OR youth* OR “young people”)) AND (validity OR validation OR reliable OR reliability OR accuracy)
Cochrane Library	diet* OR nutrition OR eating* OR food*) AND (assessment* OR evaluation* OR measurement*) AND (energy) in All Text AND child* OR “young person” OR youth* OR “young people” in All Text AND validity OR validation OR reliable OR reliability OR accuracy in All Text
SCOPUS	(TITLE-ABS-KEY ((diet* OR nutrition OR eating* OR food*) AND (assessment* OR evaluation* OR measurement*) AND (energy)) AND TITLE-ABS-KEY (child* OR “young person” OR youth* OR “young people”) AND TITLE-ABS-KEY (validity OR validation OR reliable OR reliability OR accuracy))
Embase	(diet* OR ‘nutrition’/exp OR nutrition OR eating* OR food*) AND (assessment* OR evaluation* OR measurement*) AND (energy) AND (child* OR ‘young person’ OR youth* OR ‘young people’/exp OR ‘young people’) AND (‘validity’/exp OR validity OR ‘validation’/exp OR validation OR reliable OR ‘reliability’/exp OR reliability OR ‘accuracy’/exp OR accuracy)

**Table 2:** Summary of databases searched, and total number of articles pulled (prior to duplicate removal).

Database	Search 1 (Oct 2024)	Search 2 (Nov 2025)	Total
PubMed	890	77	967
Embase	1136	70	1206
Web of Science (WoS)	1399	33	1432
CINAHL (EBSCO)	881	0	881
CABI (WoS)	633	11	644
Education Source (EBSCO)	38	0	38
ERIC (EBSCO)	4	0	4
Cochrane Library	141	0	141
SCOPUS	718	66	784
Total	5840	257	6097

**Table 4:** Support tools used by age group (*n* = 56).

Author, Study Year	Provided Standardized Household Items ^a^	Used/ Measured Household Items	Visual Aids ^b^	Food Models	Forgotten Foods List	Samples of Food Displayed	Rulers	Direct Reading of Food Packaging or Direct Weighing	Food Photographs	Did not Include or Describe Support Tools
Study samples including only children ≤5 years (*n* = 7)										
Bailey, 2018 [21] *										X
Barkin, 2018 [22]										X
BayindirGümüş, 2020 [23] *			X							
Davison, 2013 [26]										X
Eldridge, 2019 [29] *	X		X		X					
Ip, 2018 [33]		X	X							
Ruel, 2006 [68]										X
Study samples including only children 6–12 years (*n* = 20)										
Al-Kutbe, 2017 [19]			X							
Andersen, 2014 [20]										X
Ball, 2005 [61]										X
da Silva, 2021 [25]	X									
Deierlein, 2014 [27]										X
Epstein, 2021 [30]			X							
Epstein, 2005 [67]	X			X			X			
Epstein, 2002 [66]	X						X			
Evans, 2016 [31]										X
Hart, 2022 [32]			X							
Author, study year	Provided standardized household items ^a^	Used/ measured household items	Visual aids ^b^	Food models	Forgotten foods list	Samples of food displayed	Rulers	Direct reading of food packaging/ direct weighing	Food photographs	Did not include or describe support tools
Hassapidou, 2009 [53]										X
Kral, 2016 [36]			X							
Lahoz-Garcia, 2015 [37]										X
Lopez-Frias, 2005 [57]										X
Luo, 2021 [40]										X
Overcash, 2019 [42]	X		X	X						
Reed, 2013 [43]			X							
Saelens, 2018 [45]				X						
Sharkey, 2012 [46]		X	X	X						
Yang, 2017 [48]		X						X	X	
Study samples including children 6 years and older (*n* = 10)										
Arija, 2010 [64]			X							
Coufopoulos, 2003 [62]			X							
Jamshidnia, 2021 [34]			X							
Jildeh, 2011 [54]			X							
Lee, 2020 [38]										X
Li, 2017 [39]										X
Magkos, 2006 [58]	X			X						
Morais, 2024 [41]			X					X		
Sonneville, 2008 [63]										X
Zakarneh, 2023 [49]				X						
Mixed-age samples including children between 0 and 18 years, not included elsewhere (*n* = 15)										
Bernabe-Garcia, 2019 [24]				X						
Bornhorst, 2014 [12]			X							
Author, study year	Provided standardized household items ^a^	Used/ measured household items	Visual aids ^b^	Food models	Forgotten foods list	Samples of food displayed	Rulers	Direct reading of food packaging/ direct weighing	Food photographs	Did not include or describe support tools
Campbell, 2006 [51]										X
Elbel, 2015 [28]	X									
Elliott, 2011 [52]	X						X			
Hendrie, 2011 [65]				X						
Kolahdooz, 2016 [35]	X			X	X					
Labadarios, 2005 [55]	X			X		X				
Lioret, 2009 [56]			X							
Roche, 2007 [59]		X				X		X		
Rohde, 2017 [44]			X							
Sadik, 2010 [69]				X						
Serra-Majem, 2021 [60]		X								
Shiau, 2017 [47]			X							
Zekovic, 2022 [50]			X							

Notes. X Indicates the type of support was described in the study. * Indicates studies that included children <2 years (Bailey et al. [21], BayindirGumus et al. [23] and Eldridge et al. [29]). ^a^. Provided standardized items: includes measuring cups, spoons, glasses, bowls, etc. ^b^. Visual aids: includes 2-dimensional reference books (with photos of portion sizes, items), food photograph albums, USDA food amounts booklet, etc.

**Table 5:** Summary of proxy reporting by age group (*n* = 52).

Author, Study Year	Single-Proxy Reporting	Child-Assisted Proxy Reporting ^a^	Consensus Recall Approach	Multiple-Proxy Reporting	Unclear if Proxy Reporting Used	Did Not Use or Describe Proxy Reporting
Study samples including only children ≤5 years (*n* = 9)						
Bailey, 2018 [21] *	X					
Barkin, 2018 [22]	X					
BayindirGümüş, 2020 [23] *	X					
Davison, 2013 [26]	X					
Eldridge, 2019 [29] *	X					
Ip, 2018 [33]				X		
Author, study year	Single-Proxy Reporting	Child-Assisted Proxy Reporting	Consensus Recall Approach	Multiple-Proxy Reporting	Unclear if Proxy Reporting Used	Did Not Use or Describe Proxy Reporting
Ruel, 2006 [68]				X		
Sadik, 2010 [69] ^b^	X					
Serra-Majem, 2021 [60] ^b^				X		
Study samples including only children 6–12 years (*n* = 20)						
Al-Kutbe, 2017 [19]						X
Andersen, 2014 [20]						X
Ball, 2005 [61]						X
da Silva, 2021 [25]						X
Deierlein, 2014 [27]	X					
Epstein, 2021 [30]	X					
Epstein, 2005 [67]						X
Epstein, 2002 [66]		X				
Evans, 2016 [31]				X		
Hart, 2022 [32]						X
Hassapidou, 2009 [53]						X
Kral, 2016 [36]	X					
Lahoz-Garcia, 2015 [37]						X
Lopez-Frias, 2005 [57]				X		
Luo, 2021 [40]						X
Overcash, 2019 [42]						X
Reed, 2013 [43]						X
Saelens, 2018 [45]			X			
Sharkey, 2012 [46]						X
Yang, 2017 [48]						X
Study samples included children 6 years and older (*n* = 10)						
Arija, 2010 [64]		X				
Coufopoulos, 2003 [62]						X
Jamshidnia, 2021 [34]		X				
Jildeh, 2011 [54]						X
Lee, 2020 [38]	X					
Li, 2017 [39]					X	
Magkos, 2006 [58]						X
Morais, 2024 [41]						X
Sonneville, 2008 [63]						X
Zakarneh, 2023 [49]	X					
Mixed-age samples including children between 0 and 18 years, not included elsewhere (*n* = 13)						
Bernabe-Garcia, 2019 [24]					X	
Bornhorst, 2014 [12]				X		
Campbell, 2006 [51]	X					
Elbel, 2015 [28]	X					
Author, study year	Single-Proxy Reporting	Child-Assisted Proxy Reporting	Consensus Recall Approach	Multiple-Proxy Reporting	Unclear if Proxy Reporting Used	Did Not Use or Describe Proxy Reporting
Elliott, 2011 [52]						X
Hendrie, 2011 [65] ^c^		X				
Kolahdooz, 2016 [35] ^c^	X					
Labadarios, 2005 [55]	X					
Lioret, 2009 [56] ^c^		X				
Roche, 2007 [59]	X					
Rohde, 2017 [44]	X					
Shiau, 2017 [47]		X				
Zekovic, 2022 [50]	X					

**Notes.** X Indicates the type of support was described in the study. * Indicates studies that included children <2 years (Bailey et al. [21], BayindirGümüş et al. [23] and Eldridge et al. [29]). ^a^. Child-assisted proxy reporting: includes instances where the parent/caregiver was the reporter, and the child offered assistance either during the assessment or during follow-up interviews. ^b^ Sadik et al. [69]’s study sample included children 2–12 years; however, specified proxy reporting was only used for children 2–5 years; Serra-Majem et al. [60]’s study sample included children 2–13 years; however, specified proxy reporting was only used for children 2–5 years. ^c^ Hendrie et al. [65]’s study sample included children 4–13 years—proxy reporting for children <10 years; Kolahdooz et al. [35]’s study sample included children 5–13 years, specified proxy reporting was used for children <8 years; Lioret et al. [56]’s study sample included children 3–14 yrs; specified proxy reporting was used for children <10 years.

**Table 6:** Summary of dietary misreporting methods and prevalences by age group (*n* = 52).

Author, Study Year	Black Criteria	Goldberg Criteria	Comparisons to BMR, EER, or TEE	Plausibility Cutoffs ^a^	Unclear Which Method Used	Prevalence	Did Not Use or Describe Dietary Misreporting Methods
Study samples including only children ≤5 years (*n* = 7)							
Bailey, 2018 [21] *							X
Barkin, 2018 [22]							X
BayindirGümüş, 2020 [23] *							X
Davison, 2013 [26]							X
Eldridge, 2019 [29] *							X
Ip, 2018 [33]							X
Ruel, 2006 [68]							X
Study samples including only children aged 6–12 years (*n* = 20)							
Al-Kutbe, 2017 [19]			X			UR: *n* = 27 (19.9%)	
Andersen, 2014 [20]			X			UR: 9–14%; OR: 2–3%	
Ball, 2005 [61]							X
da Silva, 2021 [25]				X		UR: *n* = 2; OR: *n* = 16	
Deierlein, 2014 [27]				X		UR: *n* = 23; OR: *n* = 1 excluded	
Epstein, 2021 [30]			X			UR: 39% of sample excluded	
Epstein, 2005 [67]							X
Epstein, 2002 [66]							X
Evans, 2016 [31]				X		OR: *n* = 37 excluded (1.4%)	
Hart, 2022 [32]							X
Hassapidou, 2009 [53]							X
Kral, 2016 [36]							X
Lahoz-Garcia, 2015 [37]							X
Lopez-Frias, 2005 [57]							X
Luo, 2021 [40]				X		OR: *n* = 2	
Overcash, 2019 [42]				X		*n* = 29 (individual recall days)	
Reed, 2013 [43]							X
Saelens, 2018 [45]							X
Author, study year	Black Criteria	Goldberg Criteria	Comparisons to BMR, EER, or TEE	Plausibility Cutoffs ^a^	Unclear which method used	Prevalence	Did not use or describe dietary misreporting methods
Sharkey, 2012 [46]							X
Yang, 2017 [48]	X					*n* = 95/236 excluded (~40%)	
Study samples including children 6 years and older (*n* = 10)							
Arija, 2010 [64]							X
Coufopoulos, 2003 [62]			X			*n* = 44 (42% of original sample)	
Jamshidnia, 2021 [34]					X	*n* = 18	
Jildeh, 2011 [54]							X
Lee, 2020 [38]							X
Li, 2017 [39]							X
Magkos, 2006 [58]							X
Morais, 2024 [41]		X				Boys: UR: *n* = 63; OR: *n* = 4 Girls: UR: *n* = 45; OR: *n* = 5	
Sonneville, 2008 [63]				X		Did not describe	
Zakarneh, 2023 [49]							X
Mixed-age samples including children between 0 and 18 years, not included elsewhere (*n* = 15)							
Bernabe-Garcia, 2019 [24]							X
Bornhorst, 2014 [12]	X	X				UR: *n* = 1150 (9.5%) OR: *n* = 407 (3.4%)	
Campbell, 2006 [51]				X		OR: *n* = 7	
Elbel, 2015 [28]							X
Elliott, 2011 [52]			X ^b^			45% of sample excluded	
Hendrie, 2011 [65]							X
Kolahdooz, 2016 [35]				X		*n* = 2 excluded	
Labadarios, 2005 [55]							X
Lioret, 2009 [56]				X		UR: *n* = 5 excluded	
Roche, 2007 [59]							X
Rohde, 2017 [44]							X
Sadik, 2010 [69]							X
Serra-Majem, 2021 [60]			X			Did not describe	
Shiau, 2017 [47]							X
Zekovic, 2022 [50]							X

**Notes:** X Indicates the type of support was described in the study. Abbreviations: BMR: basal metabolic rate; EER: estimated energy requirements; OR: over-reports; TEE: total energy expenditures; UR: under-reports. * Indicates studies that included children <2 years (Bailey et al. [21], BayindirGumus et al. [23], and Eldridge et al. [29]). ^a^ Plausibility cutoffs include studies that defined minimum and maximum kcal cutoffs or that used comparisons between +/− SD from the mean. ^b^ Elliott et al. [52] used McCrory et al. [72]’s approach comparing reported energy intake with predicted TEE.

## Data Availability

All data underlying the results are available within the article and its Supplementary Materials. No additional source data are required.

## Supplementary Materials

The following supporting information can be downloaded at https://www.mdpi.com/article/10.3390/nu18213631/s1. Data Extraction Template—Scoping Review.

## Abbreviations

The following abbreviations are used in this manuscript:

BMR	Basal metabolic rate
EER	Estimated energy requirements
EI	Energy intake
FFQ	Food frequency questionnaire
FR	Food record
OR	Over-report
PRISMA-ScR	Preferred Reporting Items for Systematic Review and Meta-Analyses extension for Scoping Review
TEE	Total energy expenditure
UR	Under-report
US	United States
24HR	24 h dietary recall
