# Supplementary material for: Methods Used to Support Dietary Assessment Accuracy in Young Children: A Scoping Review
Source: Nutrients. 2026 Oct 31;18(21):3631. doi: 10.3390/nu18213631 (PMC13765226; doi:10.3390/nu18213631)
Supplement: Supplementary file 1 [file nutrients-18-03631-s001.zip › nutrients-4640485-supplementary.pdf]

Data Extraction Template – Scoping Review

General Information

- Study ID
- Title
- Year of Publication
- Lead Author Contact Details
- Country in Which the Study was Conducted
    - United States
    - UK
    - Canada
    - Australia
    - Other: ________________

Characteristics of Included Studies

- Study Aim
- Study Design
- Start Date
- End Data
- Study Funding Sources
- Possible conflicts of Interest for study authors

Dietary Assessment Characteristics

- Type of dietary assessment used:
    - 24-hour dietary recall (24HR)
    - Food frequency questionnaire (FFQ)
    - Food record (FR)
    - Other: _______________
- What setting was the assessment conducted in?
    - Free-living
    - School-based
    - Clinic based
    - Other: ________________
- How many days of assessment were included: _____#_____
- Did the assessment include both weekdays and weekend days?
    - Yes
    - No

    - Unsure, did not describe
- What was the modality of the assessment?
    - Written
    - Interview-administered
    - Web-based
    - Other:_________________________

- Did the authors mention any specific training for the dietary assessment?
    - Yes
    - No
- If yes, describe the training:
  ____
  ____

- Did the authors mention the assessment tool was validated?
    - Yes
    - No
- If yes to above, was a description of how the tool/measure was validated included in the manuscript?
    - Yes
    - No
    - Not applicable
- If yes to above, describe how the tool/measure was validated:
  ____
  ____

- Was technology used in the assessment?
    - Yes
    - No
- If yes, describe how technology was used:
  ____
  ____
- Were additional tools (i.e., visual aids, portion size estimation books, household utensils, etc.) used to help enhance the accuracy of dietary assessment?
    - Yes
    - No
- If yes, describe which tools were used and how these tools were used:
  ____
  ____
- Was proxy-reporting used?
    - Yes

- No

- If yes, describe how proxy-reporting was used:

  ____

  ____

- Was there mention of how dietary misreporting would be handled?
  - Yes
  - No
  - Not applicable
- If yes, describe how dietary misreporting was handled.

  ____

  ____

- Were findings related to misreporting discussed, if so, describe (i.e., prevalence of misreporting):

  ____

  ____

Additional Details

- Population description
- Study Inclusion Criteria
- Study Exclusion Criteria
- Method of Recruitment of study participants
  - Phone
  - Mail
  - Clinic Patients
  - Volunteers
  - Other: ______________________
- Total sample size: _______#____________

Child Characteristics

- Age group
  - Toddler (2 years)
  - Preschoolers (3-5 years)
  - School-aged children (6-12 years)
- Additional information about children (i.e., disease-specific states, medical conditions, etc.)

Parent Characteristics

- Age group (years)
  - 18-29
  - 30-39

    - 40-49
    - 50-59
    - 60+
    - did not report
    - not applicable
- Person responsible for proxy reporting (if applicable)
    - Mother
    - Father
    - Other guardian
    - Grandparent(s)
    - Did not disclose
    - Not applicable
    - Other: __________
